# Effects of probiotic or synbiotic supplementation on glycolipid metabolism in patients with coronary heart disease: a systematic review and meta-analysis

**DOI:** 10.3389/fmicb.2026.1877366

**Published:** 2026-10-23

**Authors:** Yanan Bai, Yunfeng Yu, Xiangning Huang, Xinyu Yang, Rong Yu, Qin Xiang

**Affiliations:** 1 School of Traditional Chinese Medicine, Hunan University of Chinese Medicine, Changsha, Hunan, China; 2 Department of Endocrinology, The First Hospital of Hunan University of Chinese Medicine, Changsha, Hunan, China

**Keywords:** coronary heart disease, glycolipid metabolism, meta-analysis, probiotic, synbiotic, systematic review

## Abstract

**Objective:**

This systematic review and meta-analysis, combined with trial sequential analysis (TSA), evaluated the effects of probiotic/synbiotic supplementation on glycolipid metabolism in patients with coronary heart disease (CHD).

**Methods:**

Randomized controlled trials published up to May 1, 2026, were systematically searched across major databases, including PubMed, Embase, Cochrane Library, Web of Science, MEDLINE, and ClinicalTrials.gov. Pooled analyses were performed using Review Manager 5.3, and TSA was conducted to assess the conclusiveness of evidence. Mean difference (MD) and risk ratio (RR) were calculated for continuous and dichotomous outcomes, respectively.

**Results:**

Six trials involving 296 participants were included. Probiotic/synbiotic supplementation was associated with a reduction in fasting blood glucose (FBG) (MD − 19.25 mg/dL, 95% CI −32.28 to −6.22, *p* = 0.004) and an increase in high-density lipoprotein cholesterol (HDL-C) (MD 3.70 mg/dL, 95% CI 1.65 to 5.74, *p* = 0.0004) based on four studies involving 192 participants. No significant effects were observed on low-density lipoprotein cholesterol, very low-density lipoprotein cholesterol, total cholesterol, triglycerides, insulin, homeostasis model assessment of insulin resistance, quantitative insulin sensitivity check index, systolic blood pressure, or diastolic blood pressure (*p* ≥ 0.05). Although adverse event rates did not differ significantly (*p* ≥ 0.05), safety evidence was limited by few events and a wide confidence interval. TSA showed that the cumulative evidence for both HDL-C and FBG did not cross the prespecified monitoring boundaries or reach the required information sizes. The certainty of evidence for all outcomes was rated as low or very low.

**Conclusion:**

Probiotic/synbiotic supplementation may be associated with increased HDL-C and reduced FBG levels in patients with CHD. However, given the negative TSA findings, low certainty of evidence, and lack of hard cardiovascular outcomes, changes in HDL-C and FBG cannot be regarded as direct evidence of cardiovascular benefit. Furthermore, generalizability is limited, as five of the six studies were conducted in Iran and only one in China, which may limit applicability to populations with different dietary patterns, microbiome profiles, ethnic backgrounds, or healthcare systems. The substantial heterogeneity in probiotic strains, formulations, doses, and treatment durations also limits generalization to probiotics or synbiotics as a whole.

**Systematic review registration:**

https://www.crd.york.ac.uk/PROSPERO/view/CRD420251178187; identifier: CRD420251178187.

## Graphical abstract

**Figure:**
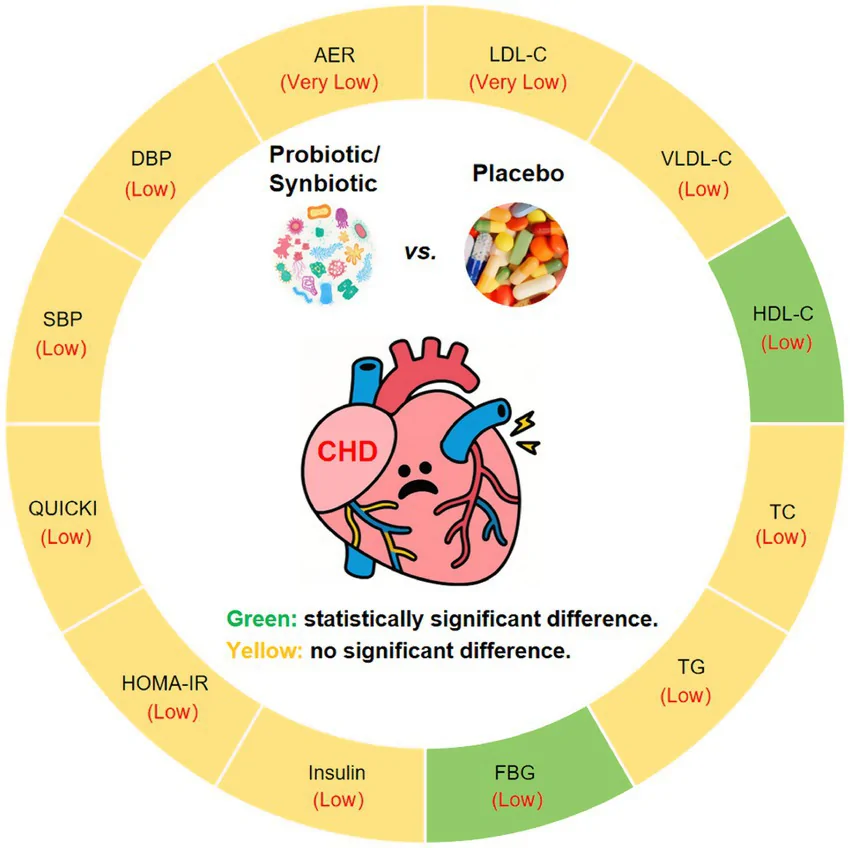
LDL-C, low-density lipoprotein cholesterol; VLDL-C, very low-density lipoprotein cholesterol; HDL-C, high-density lipoprotein cholesterol; TC, total cholesterol; TG, triglycerides; FBG, fasting blood glucose; HOMA-IR, homeostasis model assessment of insulin resistance; QUICKI, quantitative insulin sensitivity check index; SBP, systolic blood pressure; DBP, diastolic blood pressure; AER, adverse event rate.

## 1 Introduction

Coronary heart disease (CHD) is a chronic ischemic condition caused by atherosclerotic obstruction of the coronary arteries and remains the leading cause of cardiovascular mortality worldwide (Vrints et al., 2024; World Health Organization, n.d.). Its pathogenesis involves lipid infiltration, endothelial dysfunction, oxidative stress, and inflammation (Sayols-Baixeras et al., 2014; Jebari-Benslaiman et al., 2022). Among these, abnormal lipid metabolism plays a central role in atherosclerotic plaque formation and progression (Yu et al., 2013; Allahverdian et al., 2014). Although conventional treatments such as antithrombotic, antihypertensive, and lipid-lowering therapies have improved patient outcomes, their use may be limited by treatment-related adverse effects including bleeding, cough, angioedema, and statin-associated muscle symptoms (Kalyanasundaram et al., 2011; Bots et al., 2022; Zeng et al., 2025). Therefore, identifying safe and effective adjunct therapies to improve prognosis in patients with CHD is of great clinical importance.

Emerging evidence suggests that gut microbiota dysbiosis contributes to cardiovascular diseases by altering host metabolism and inflammatory pathways (Rahman et al., 2022). Patients with CHD exhibit significant alterations in gut microbial composition, which may influence host metabolism and inflammation through metabolites such as p-cresol sulfate and phenylacetylglutamine (Talmor-Barkan et al., 2022). Since disturbances in glucose and lipid metabolism are considered important mediators linking gut microbiota to CHD, probiotic/synbiotic supplementation has attracted growing attention as a potential therapeutic approach (Roessler et al., 2022). Several recent meta-analyses have consistently suggested that probiotic/synbiotic supplementation may improve glycolipid metabolism in patients with CHD (Zhong et al., 2026; Yang et al., 2025; Lei et al., 2023; Dong et al., 2025). However, the earlier meta-analyses by Lei et al. (2023) and Dong et al. (2025) included trials in which probiotics or synbiotics were co-administered with vitamin D or selenium, making it difficult to determine the independent effects of probiotic/synbiotic supplementation. In contrast, the more recent meta-analyses by Zhong et al. (2026) and Yang et al. (2025) included studies involving patients with acute myocardial infarction, which may have introduced additional clinical heterogeneity when evaluating glycolipid metabolic outcomes. Furthermore, these studies did not apply trial sequential analysis (TSA) to control for random errors, which may limit the robustness of their conclusions.

To address these limitations, we performed an updated meta-analysis combined with TSA to further evaluate the effects of probiotic/synbiotic supplementation on glycolipid metabolism in patients with CHD. We hypothesized that probiotic/synbiotic supplementation would improve glucose and lipid parameters, thereby providing further evidence regarding its potential as an adjunctive therapy for CHD management.

## 2 Methods

This meta-analysis was performed following the guidelines established by the Preferred Reporting Items for Systematic Reviews and Meta-Analyses (PRISMA) (Page et al., 2021). The protocol was registered in the International Prospective Register of Systematic Reviews (PROSPERO, registration number: CRD420251178187). The initial search was conducted on May 1, 2025, and the updated search was conducted on May 1, 2026.

### 2.1 Inclusion and exclusion criteria

Inclusion criteria: (i) Patients with a diagnosis of CHD established according to accepted clinical criteria, including a documented history of coronary atherosclerotic disease confirmed by clinical assessment, relevant medical history, electrocardiographic findings, coronary angiography, or other recognized diagnostic modalities, as reported in the original studies. No restrictions were imposed on gender, age, ethnicity, or disease duration. (ii) Interventions: The experimental group received probiotic/synbiotic supplementation in addition to standard treatment for CHD. Synbiotics were included because they contain probiotics combined with prebiotics that support probiotic viability and function. (iii) Comparisons: The control group received a placebo in addition to standard treatment for CHD. The standard treatment for both the experimental and control groups included antiplatelet agents, statins for lipid lowering, and dietary management. For patients with coexisting type 2 diabetes mellitus (T2DM), insulin therapy was also administered as part of their treatment. (iv) Outcomes: Lipid metabolism outcomes included LDL-C, very low-density lipoprotein cholesterol (VLDL-C), high-density lipoprotein cholesterol (HDL-C), TC, and TG. Glucose metabolism outcomes included FBG, insulin, homeostasis model assessment of insulin resistance (HOMA-IR), and quantitative insulin sensitivity check index (QUICKI). Blood pressure outcomes included systolic blood pressure (SBP) and diastolic blood pressure (DBP). The safety outcome was the adverse event rate (AER). Adverse event data were extracted as reported in the original trials. When available, information on the number, type, severity, relatedness, and ascertainment methods of adverse events was extracted from the original reports. (v) Study design: Randomized controlled trials (RCTs) only.

Exclusion criteria: (i) Studies involving patients with newly diagnosed myocardial infarction; (ii) Duplicate publications or retracted articles; (iii) Publications with unavailable full text or incomplete data; (iv) Publications with obvious statistical errors.

### 2.2 Literature search strategy

A comprehensive literature search was performed across six electronic databases: PubMed, Embase, Cochrane Library, Web of Science, MEDLINE, and ClinicalTrials.gov. The first five databases were searched for published studies, whereas ClinicalTrials.gov was searched for completed, unpublished, or ongoing trials. The search strategy combined Medical Subject Headings (MeSH) and free-text terms in the title or abstract, including: (Coronary Disease OR Coronary Diseases OR Coronary Heart Disease OR Coronary Heart Diseases OR Coronary Artery Disease OR Coronary Artery Diseases OR Myocardial Infarction OR Heart Attack OR Heart Attacks OR Myocardial Infarct OR Myocardial Infarcts) AND (Probiotic OR Probiotics OR Synbiotics OR Synbiotic OR Bifidobacterium OR Bifidobacteria OR Bacillus bifida OR Yeast OR *Saccharomyces cerevisiae* OR Saccharomyces italicus OR Saccharomyces oviformis OR S cerevisiae OR *S. cerevisiae* OR Saccharomyces uvarum var. melibiosus OR *Candida robusta* OR Saccharomyces capensis OR *Lactobacillus acidophilus* OR *Lactobacillus amylovorus* OR Lactobacill OR lactic acid bacteria OR *Clostridium butyricum* OR Bacillus OR Natto Bacteria OR Streptococcus thermophiles OR Enterococcus). Myocardial infarction-related terms were retained in the search strategy to maximize retrieval sensitivity because CHD-related trials may be indexed or described using myocardial infarction terminology. During study selection, however, trials enrolling patients with acute or newly diagnosed myocardial infarction were excluded according to the predefined eligibility criteria. The search spanned from database inception through May 1, 2026, with no language restrictions. Detailed search strategies and the number of records for each database are provided in Supplementary Table S1. Additionally, reference lists of relevant reviews and included articles were manually screened to ensure comprehensive coverage.

### 2.3 Study selection process

All identified records were imported into EndNote X9 software, where duplicates were automatically removed and manually verified. Two independent reviewers assessed the titles and abstracts based on the predefined inclusion and exclusion criteria. Full-text articles of potentially eligible studies were retrieved for further evaluation by both reviewers. Discrepancies were resolved through discussion or by consulting a third reviewer. The PRISMA flow diagram was utilized to depict the study selection process.

### 2.4 Data extraction

Data were extracted independently by two reviewers using a standardized form, capturing information such as authorship, publication year, country, sample size, demographic details, intervention specifics, control conditions, measured outcomes, funding sources, and conflicts of interest. Any discrepancies were discussed to reach consensus. If completed but unpublished trials had been identified through ClinicalTrials.gov, investigators would have been contacted when relevant outcome data were not publicly available. Corresponding authors were also contacted when necessary to clarify missing or unclear data.

### 2.5 Risk of bias assessment

The risk of bias in the included randomized controlled trials was independently assessed by two reviewers using the Cochrane Risk of Bias 2 (RoB 2) tool. The assessment covered five domains: (i) bias arising from the randomization process; (ii) bias due to deviations from intended interventions; (iii) bias due to missing outcome data; (iv) bias in measurement of the outcome; and (v) bias in selection of the reported result. Each domain was judged as low risk of bias, some concerns, or high risk of bias according to the RoB 2 guidance. An overall risk-of-bias judgment was derived for each outcome based on the domain-level assessments. Two reviewers independently performed the assessments, and disagreements were resolved through discussion or consultation with a third reviewer.

### 2.6 Data synthesis and statistical analysis

Meta-analyses were conducted using Review Manager 5.3 software. For dichotomous outcomes, risk ratios (RRs) with 95% confidence intervals (CIs) were calculated. For continuous outcomes, mean differences (MDs) with 95% CIs were calculated because the outcomes were measured using the same scales across studies. For all continuous outcomes, including LDL-C, VLDL-C, HDL-C, TC, TG, FBG, insulin, HOMA-IR, QUICKI, SBP, and DBP, post-intervention endpoint values were used for meta-analysis. All included studies reported the corresponding means and standard deviations (SDs); therefore, no conversion or imputation of SDs was required. Before pooling, laboratory measurements were converted to consistent units when necessary. Statistical heterogeneity was assessed using Cochran’s *Q* test and the *I*^2^ statistic. An *I*^2^ value of <50% was considered indicative of low statistical heterogeneity, whereas *I*^2^ ≥ 50% indicated substantial statistical heterogeneity. Given the anticipated clinical heterogeneity among the included studies, including differences in country, participant characteristics, comorbidities, probiotic strains, intervention type, dosage, treatment duration, and background treatment, a random-effects model was used for all outcomes regardless of the degree of statistical heterogeneity.

Subsequently, to assess the robustness of the meta-analysis results, a sensitivity analysis was performed. This involved systematically excluding individual studies to evaluate their impact on the overall findings. Each study was sequentially excluded to evaluate its influence on the pooled estimates, heterogeneity, and statistical significance of the results. Changes in the pooled effect estimate, confidence interval, statistical significance, or heterogeneity after exclusion of individual studies were evaluated to identify potentially unstable findings. Additionally, exploratory subgroup analyses were conducted to investigate potential sources of clinical heterogeneity and their impact on the outcomes, focusing on country, average age, intervention type, probiotic strain, probiotic dosage, and treatment duration.

Furthermore, TSA was performed using TSA 0.9.5.10 beta software to control the risk of random errors and assess the sufficiency of cumulative evidence. A two-sided alpha of 5% and a statistical power of 80% were applied, with O’Brien–Fleming-type alpha-spending monitoring boundaries. The anticipated intervention effects used for TSA were derived independently from effect estimates reported in relevant previous studies rather than from the pooled estimates of the present meta-analysis. A random-effects model was applied in accordance with the primary meta-analysis, and the required information size was calculated based on the specified parameters. When substantial statistical heterogeneity was present, the required information size was adjusted for between-study diversity using a diversity-adjusted required information size (DARIS). TSA was used to evaluate whether the accumulated information was sufficient in relation to the specified anticipated intervention effect and whether the cumulative Z-curve crossed the sequential monitoring boundaries.

### 2.7 Publication bias

Publication bias was assessed using funnel plots and Egger’s tests only when at least 10 studies were available for a given outcome. Funnel plot asymmetry was visually inspected to evaluate the potential risk of publication bias, with a symmetrical distribution indicating a lower likelihood of publication bias and an asymmetrical distribution suggesting possible publication bias. Egger’s test evaluated the relationship between the standard error of the effect estimates and the effect sizes to detect funnel plot asymmetry. A *p*-value < 0.05 was considered suggestive of potential publication bias. When fewer than 10 studies were included, publication bias was not assessed because both funnel plots and Egger’s tests have limited statistical power and reliability with a small number of studies. This approach was adopted to avoid potentially misleading interpretations arising from underpowered assessments of small-study effects and funnel plot asymmetry.

### 2.8 Certainty of evidence

The certainty of evidence for each outcome was evaluated using the Grading of Recommendations, Assessment, Development, and Evaluation (GRADE) system. This assessment considered factors such as risk of bias, inconsistency, indirectness, imprecision, and publication bias. Each outcome was classified as having high, moderate, low, or very low certainty of evidence. This classification informed the interpretation of the pooled estimates and the overall certainty of the findings.

## 3 Results

### 3.1 Study selection

The comprehensive literature search across various databases yielded a total of 2,657 records: 420 from PubMed, 647 from Embase, 144 from the Cochrane Library, 763 from Web of Science, 620 from MEDLINE, 41 from ClinicalTrials.gov, and 22 from reference lists. After eliminating 1,042 duplicate entries, 1,615 records remained for screening based on titles and abstracts. This initial screening led to the exclusion of 1,568 studies deemed irrelevant. The full texts of the remaining articles were then evaluated for eligibility, resulting in the exclusion of 41 studies: three were excluded due to duplicate data, one did not meet the diagnostic criteria for participants, 28 did not fulfill the intervention criteria, eight failed to satisfy the outcome measures, and one was retracted. Ultimately, six studies were included in this meta-analysis (Sun et al., 2022; Moludi et al., 2019; Raygan et al., 2018; Liu et al., 2024; Moludi et al., 2022; Tajabadi-Ebrahimi et al., 2017). As no completed but unpublished clinical trials meeting the predefined eligibility criteria were identified in ClinicalTrials.gov, no investigators were contacted regarding unavailable results. The detailed flow diagram is presented in Figure 1.

**Figure 1:**
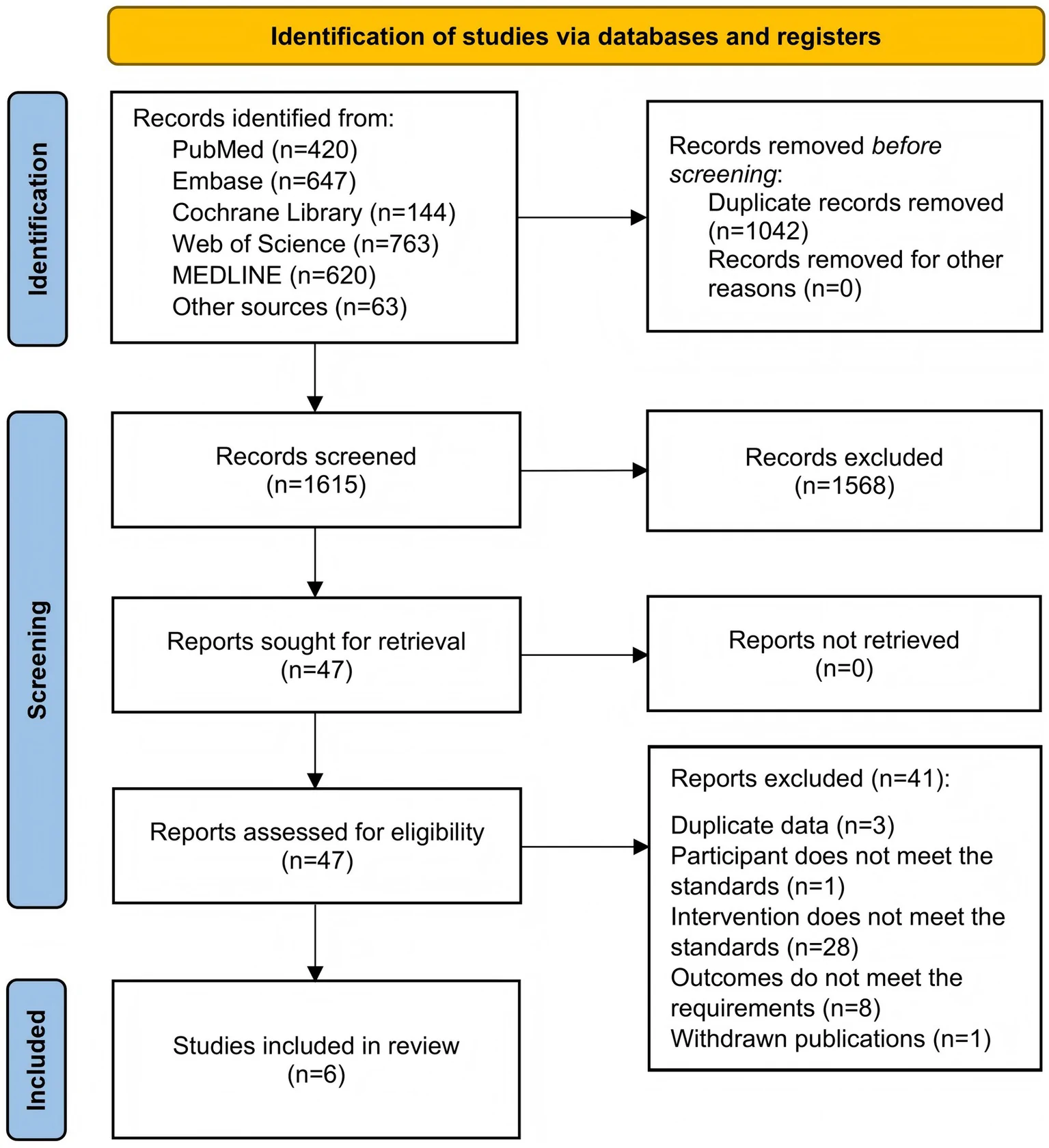
Literature screening process.

This study shares partial overlap with previous meta-analyses, with overlap rates of 50.0% with Dong et al. (2025), 62.5% with Lei et al. (2023), 75.0% with Yang et al. (2025), and 66.7% with Zhong et al. (2026). Compared with Dong et al. (2025) and Lei et al. (2023), the present study excluded trials in which probiotic/synbiotic supplementation was combined with micronutrients, such as vitamin D or selenium, thereby reducing the potential confounding effects of these co-interventions on glycolipid metabolism. Compared with Zhong et al. (2026) and Yang et al. (2025), the present study excluded the trial enrolling patients with acute myocardial infarction, thereby reducing potential clinical heterogeneity related to the acute inflammatory response, metabolic disturbances, and intensive medical management.

### 3.2 Basic characteristics

The meta-analysis incorporated six RCTs with a total of 296 participants (Sun et al., 2022; Moludi et al., 2019; Raygan et al., 2018; Liu et al., 2024; Moludi et al., 2022; Tajabadi-Ebrahimi et al., 2017). Among the studies, one was conducted in China, while the other five were based in Iran. The average age of participants was 59.6 years, with females accounting for 31.3%, and the mean BMI was 28.3 kg/m^2^. Regarding comorbidities, two studies included patients with T2DM, and one study specifically enrolled overweight or obese participants, while the remaining studies did not report major comorbid conditions. Regarding background treatment, all six studies reported concomitant standard therapies, including antiplatelet agents, statins, and dietary management, and indicated that these background treatments were comparable between the intervention and control groups. Raygan et al. (2018) and Tajabadi-Ebrahimi et al. (2017), which enrolled participants with coexisting T2DM, additionally reported insulin therapy, which was also balanced between the two groups. However, the included studies did not provide specific details about the names, doses, or treatment regimens of these concomitant medications, which limited further assessment of their potential influence on the metabolic outcomes. All interventions were administered orally in the form of probiotic/synbiotic supplements. Four studies used single-strain formulations, primarily *Lactobacillus rhamnosus* or *Bifidobacterium lactis*, whereas two studies employed multi-strain preparations containing combinations of *Lactobacillus acidophilus*, *Lactobacillus casei*, and *Bifidobacterium bifidum*. The probiotic dosage ranged from 1.6 × 10^9^ to 3 × 10^10^ CFU/day, and the treatment duration varied from 8 to 24 weeks. Additional characteristics of the included studies are summarized in Table 1.

**Table 1:** Basic characteristics of included studies.

Study	Source	Sample (E/C)	Female (%)	Age (years)	BMI (kg/m^2^)	Comorbidities	Standard treatment	Intervention	Comparison	Treatment duration (weeks)
Liu et al. (2024)	Iranian	24/12	35.4	50.5	27.2	/	/	*L. rhamnosus* 1.9 × 10^9^ CFU/d and inulin 15 mg/d	Placebo	8
Moludi et al. (2019)	Iranian	22/22	6.8	56.9	/	Overweight or obese	/	*L. rhamnosus* 1.6 × 10^9^ CFU/d	Placebo	12
Moludi et al. (2022)	Iranian	24/12	35.4	51.5	27.7	/	/	*L. rhamnosus* 1.9 × 10^9^ CFU/d	Placebo	8
Raygan et al. (2018)	Iranian	30/30	/	61.3	29.8	T2DM	Insulin	*B. bifidum*, *L. casei*, and *L. acidophilus* 2 × 10^9^ CFU/d	Placebo	12
Sun et al. (2022)	Chinese	36/24	43.3	65.6	25.3	/	/	*B. lactis* 3 × 10^10^ CFU/d	Placebo	24
Tajabadi-Ebrahimi et al. (2017)	Iranian	30/30	/	64.1	31.0	T2DM	Insulin	*L. acidophilus*, *L. casei*, and *B. bifidum* 2 × 10^9^ CFU/d and inulin 800 mg/d	Placebo	8

E, experimental group; C, control group. There were no significant differences in female ratio, average age, or disease duration between the experimental and control groups in each included study.

### 3.3 Risk of bias

The risk of bias of the six included studies was reassessed using the Cochrane RoB 2 tool, with the results presented in Figure 2.

**Figure 2:**
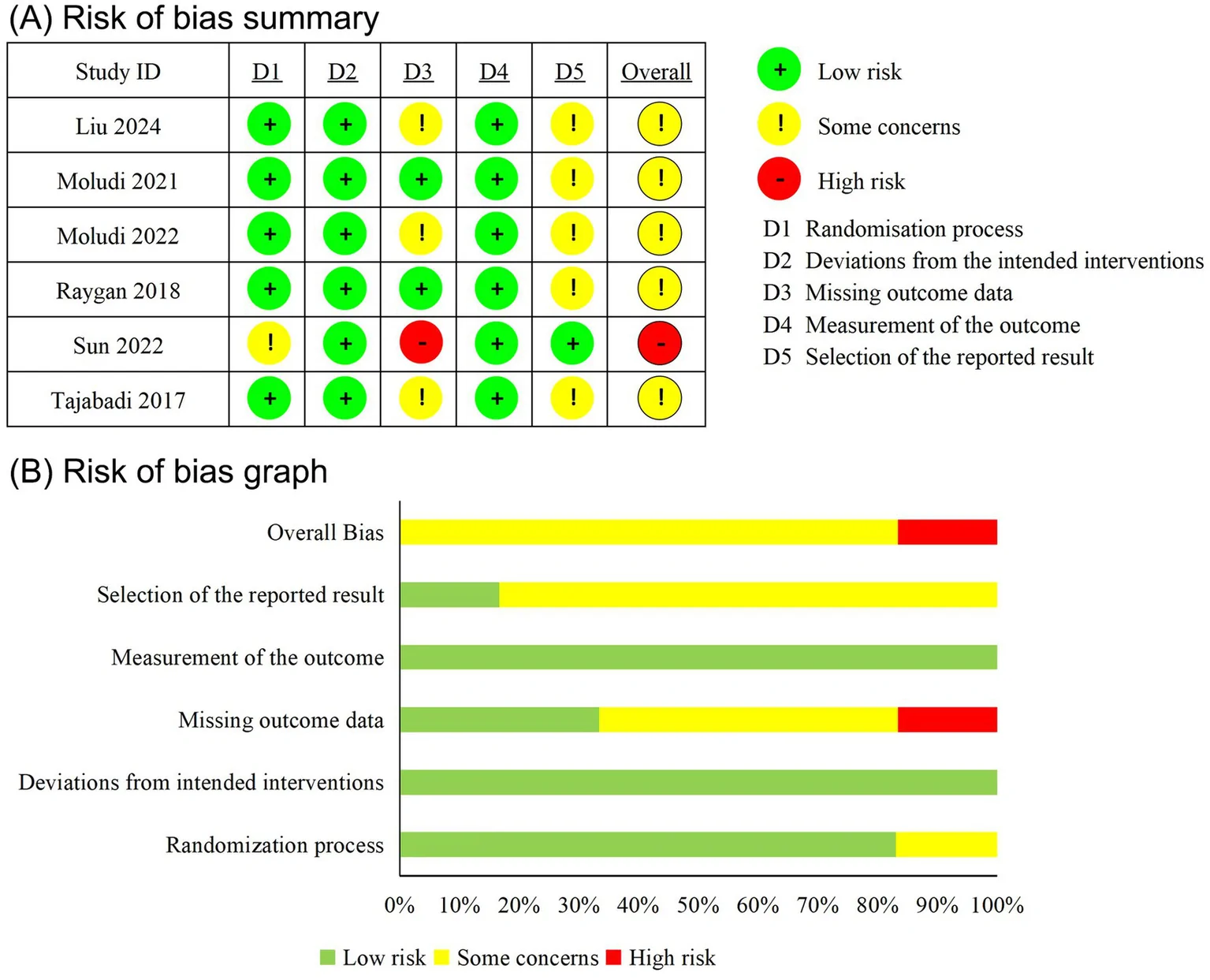
Risk assessment of bias. **(A)** Risk of bias summary. **(B)** Risk of bias graph.

For bias arising from the randomization process, five studies (Moludi et al., 2019; Raygan et al., 2018; Liu et al., 2024; Moludi et al., 2022; Tajabadi-Ebrahimi et al., 2017) were judged to be at low risk because they adequately described random sequence generation and allocation concealment. Sun et al. (2022), although reporting an appropriate randomization method, did not provide sufficient information regarding allocation concealment and was therefore judged as having some concerns in this domain.

Regarding bias due to deviations from intended interventions, all six studies (Sun et al., 2022; Moludi et al., 2019; Raygan et al., 2018; Liu et al., 2024; Moludi et al., 2022; Tajabadi-Ebrahimi et al., 2017) employed placebo-controlled, parallel-group designs and reported blinding of participants and study personnel. There was no clear evidence that deviations from the intended interventions were related to the trial context or that such deviations were likely to influence the estimated intervention effect. Therefore, all studies were judged to be at low risk in this domain.

For bias due to missing outcome data, Moludi et al. (2019) and Raygan et al. (2018) were judged to be at low risk because complete or nearly complete outcome data were available. Liu et al. (2024), Moludi et al. (2022), and Tajabadi-Ebrahimi et al. (2017) reported a small number of participant withdrawals, but the available information was insufficient to fully determine whether the missing data could have affected the study results; these studies were therefore judged as having some concerns. Sun et al. (2022) reported a dropout rate exceeding 20%, raising concerns that the missing outcome data might have influenced the estimated intervention effect, and was consequently judged to be at high risk of bias in this domain.

With respect to bias in measurement of the outcome, all studies (Sun et al., 2022; Moludi et al., 2019; Raygan et al., 2018; Liu et al., 2024; Moludi et al., 2022; Tajabadi-Ebrahimi et al., 2017) were judged to be at low risk. This judgment was based not solely on the objective nature of the outcomes, but also on the characteristics of outcome measurement reported in the individual trials. The primary outcomes, including serum lipid parameters, fasting blood glucose, insulin-related indices, and other biochemical measures, were laboratory-based continuous outcomes with prespecified quantitative measurements. Across the included trials, the outcomes were assessed using the same general type of biochemical measurements in the intervention and control groups, and no study reported differential outcome measurement procedures or changes in the measurement methods according to treatment assignment. In addition, no trial indicated that knowledge of the intervention could have substantially influenced the laboratory determination of these biochemical outcomes. Therefore, even where blinding of outcome assessors was not explicitly described, the risk that knowledge of intervention assignment affected outcome measurement was considered low.

For bias in selection of the reported result, Sun et al. (2022) was judged to be at low risk because its trial registration information was publicly accessible and the reported outcomes were consistent with the prespecified outcomes. For the remaining five studies (Moludi et al., 2019; Raygan et al., 2018; Liu et al., 2024; Moludi et al., 2022; Tajabadi-Ebrahimi et al., 2017), although trial registration numbers were reported, the corresponding registration records or sufficiently detailed prespecified analysis plans were not publicly accessible. Consequently, it was not possible to exclude the possibility of selective reporting among multiple eligible outcome measurements or analyses, and these studies were judged as having some concerns in this domain.

Overall, five studies (Moludi et al., 2019; Raygan et al., 2018; Liu et al., 2024; Moludi et al., 2022; Tajabadi-Ebrahimi et al., 2017) were judged to have some concerns regarding risk of bias, primarily because of insufficient information on the randomization process, missing outcome data, or the selection of reported results. One study (Sun et al., 2022) was judged to be at high overall risk of bias because of the substantial proportion of missing outcome data.

### 3.4 Meta-analysis

#### 3.4.1 Lipid metabolism outcomes

LDL-C: The meta-analysis for LDL-C levels included five studies (Sun et al., 2022; Raygan et al., 2018; Liu et al., 2024; Moludi et al., 2022; Tajabadi-Ebrahimi et al., 2017) involving 252 participants. The I^2^ statistic indicated high heterogeneity (I^2^ = 67%). The pooled results showed no statistically significant difference in LDL-C levels between the probiotic/synbiotic supplementation and placebo groups (MD − 9.50 mg/dL, 95% CI − 19.17 to 0.17, *p* = 0.05), as shown in Figure 3A.

**Figure 3:**
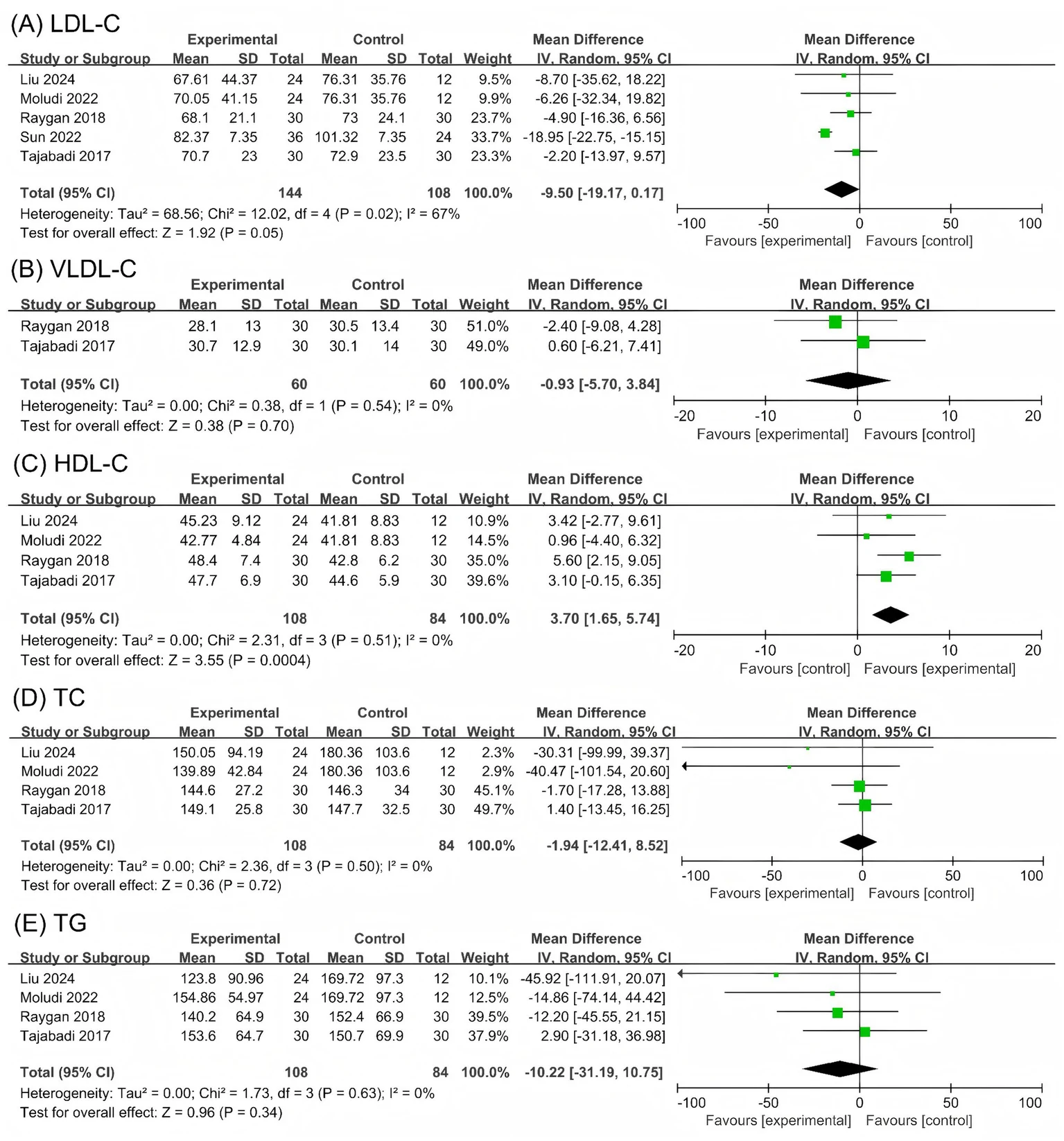
Forest plots of the meta-analysis on blood lipid outcomes: **(A)** LDL-C; **(B)** VLDL-C; **(C)** HDL-C; **(D)** TC; **(E)** TG. LDL-C, low-density lipoprotein cholesterol; VLDL-C, very low-density lipoprotein cholesterol; HDL-C, high-density lipoprotein cholesterol; TC, total cholesterol; TG, triglycerides.

VLDL-C: The meta-analysis for VLDL-C levels included two studies (Raygan et al., 2018; Tajabadi-Ebrahimi et al., 2017) involving 120 participants. The *I*^2^ statistic indicated no significant heterogeneity (*I*^2^ = 0). The pooled results indicated no statistically significant difference in VLDL-C levels between the two groups (MD − 0.93 mg/dL, 95% CI − 5.70 to 3.84, *p* = 0.70), as illustrated in Figure 3B.

HDL-C: The meta-analysis for HDL-C levels included four studies (Raygan et al., 2018; Liu et al., 2024; Moludi et al., 2022; Tajabadi-Ebrahimi et al., 2017) involving 192 participants. The I^2^ statistic indicated no significant heterogeneity (I^2^ = 0). The pooled results demonstrated that probiotic/synbiotic supplementation was associated with a significant increase in HDL-C levels compared to the placebo (MD 3.70 mg/dL, 95% CI 1.65 to 5.74, *p* = 0.0004), as depicted in Figure 3C.

TC: The meta-analysis for TC levels included four studies (Raygan et al., 2018; Liu et al., 2024; Moludi et al., 2022; Tajabadi-Ebrahimi et al., 2017) involving 192 participants. The *I*^2^ statistic indicated minor heterogeneity (*I*^2^ = 0%). The pooled results showed no significant difference in TC levels between the two groups (MD − 1.94 mg/dL, 95% CI − 12.41 to 8.52, *p* = 0.72), as shown in Figure 3D.

TG: The meta-analysis for TG levels included four studies (Raygan et al., 2018; Liu et al., 2024; Moludi et al., 2022; Tajabadi-Ebrahimi et al., 2017) involving 192 participants. The I^2^ statistic indicated no significant heterogeneity (*I*^2^ = 0). The pooled results revealed no statistically significant difference in TG levels between the two groups (MD − 10.22 mg/dL, 95% CI − 31.19 to 10.75, *p* = 0.34), as illustrated in Figure 3E.

#### 3.4.2 Glucose metabolism outcomes

FBG: The meta-analysis for FBG levels included four studies (Raygan et al., 2018; Liu et al., 2024; Moludi et al., 2022; Tajabadi-Ebrahimi et al., 2017) involving 192 participants. The I^2^ statistic indicated no significant heterogeneity (*I*^2^ = 0). The pooled results demonstrated that probiotic/synbiotic supplementation was associated with reduced FBG levels compared to the placebo (MD − 19.25 mg/dL, 95% CI − 32.28 to −6.22, *p* = 0.004), as depicted in Figure 4A.

**Figure 4:**
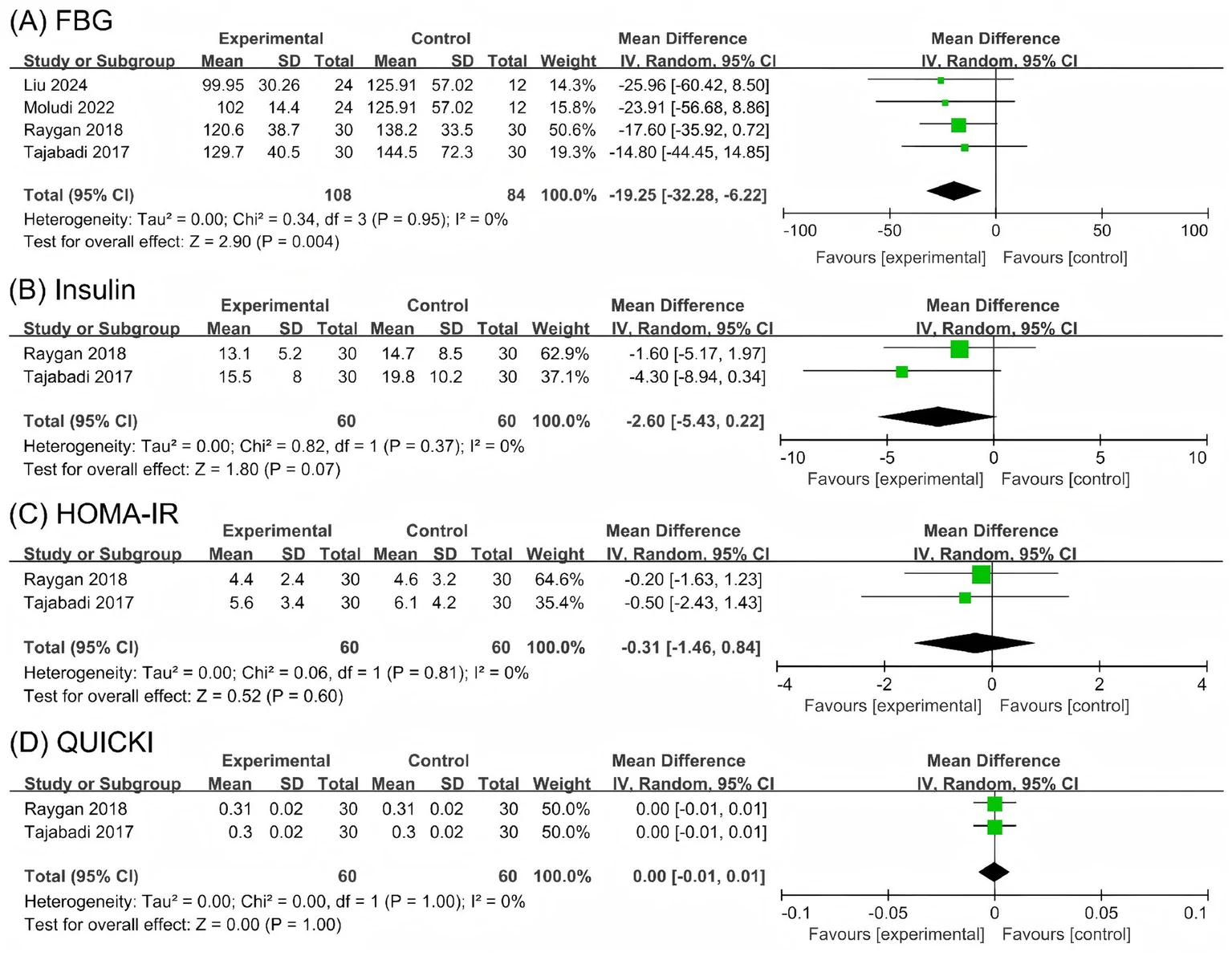
Forest plots of the meta-analysis on blood glucose outcomes: **(A)** FBG; **(B)** Insulin; **(C)** HOMA-IR; **(D)** QUICKI. FBG, fasting blood glucose; HOMA-IR, homeostatic model assessment of insulin resistance; QUICKI, quantitative insulin sensitivity check index.

Insulin: The meta-analysis for insulin levels included two studies (Raygan et al., 2018; Tajabadi-Ebrahimi et al., 2017) involving 120 participants. The *I*^2^ statistic indicated no significant heterogeneity (*I*^2^ = 0). The pooled results revealed no statistically significant difference in insulin levels between the two groups (MD − 2.60 μIU/mL, 95% CI − 5.43 to 0.22, *p* = 0.07), as shown in Figure 4B.

HOMA-IR: The meta-analysis for HOMA-IR included two studies (Raygan et al., 2018; Tajabadi-Ebrahimi et al., 2017) involving 120 participants. The *I*^2^ statistic indicated no significant heterogeneity (*I*^2^ = 0). The pooled results revealed no statistically significant difference in HOMA-IR between the two groups (MD − 0.31, 95% CI − 1.46 to 0.84, *p* = 0.60), as illustrated in Figure 4C.

QUICKI: The meta-analysis for QUICKI included two studies (Raygan et al., 2018; Tajabadi-Ebrahimi et al., 2017) involving 120 participants. The *I*^2^ statistic indicated no significant heterogeneity (*I*^2^ = 0). The pooled results revealed no statistically significant difference in QUICKI between the two groups (MD 0.00, 95% CI − 0.01 to 0.01, *p* = 1.00), as depicted in Figure 4D.

#### 3.4.3 Blood pressure outcomes

SBP: The meta-analysis for SBP included three studies (Raygan et al., 2018; Liu et al., 2024; Moludi et al., 2022) involving 132 participants. The I^2^ statistic indicated no significant heterogeneity (*I*^2^ = 0%). The pooled results revealed no statistically significant difference in SBP between the two groups (MD − 4.25 mmHg, 95% CI − 9.63 to 1.12, *p* = 0.12), as shown in Figure 5A.

**Figure 5:**
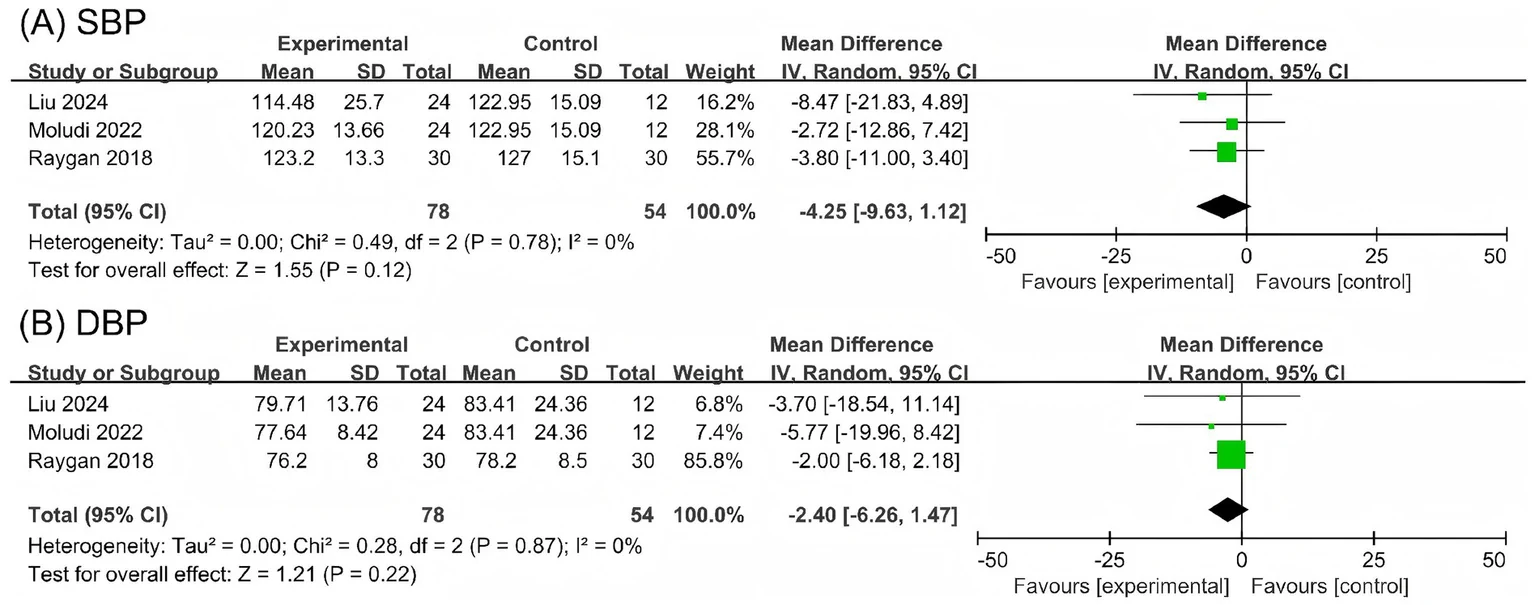
Forest plots of the meta-analysis on blood pressure outcomes: **(A)** SBP; **(B)** DBP. SBP, systolic blood pressure; DBP, diastolic blood pressure.

DBP: The meta-analysis for DBP included three studies (Raygan et al., 2018; Liu et al., 2024; Moludi et al., 2022) involving 132 participants. The I^2^ statistic indicated no significant heterogeneity (*I*^2^ = 0). The pooled results revealed no statistically significant difference in DBP between the two groups (MD − 2.40 mmHg, 95% CI −6.26 to 1.47, *p* = 0.22), as depicted in Figure 5B.

#### 3.4.4 Safety outcomes

AER: The meta-analysis for AER included two studies (Moludi et al., 2019; Raygan et al., 2018) involving 104 participants. The AER in the probiotic/synbiotic supplementation group was 3.8%, compared to 1.9% in the placebo group. The *I*^2^ statistic indicated no significant heterogeneity (*I*^2^ = 0%). The pooled analysis did not show a statistically significant difference in AER between the two groups (RR 2.00, 95% CI 0.20 to 20.49, *p* = 0.56), as shown in Figure 6. However, only two studies with a limited number of adverse events were available, resulting in a very wide confidence interval and substantial uncertainty in the pooled estimate. Furthermore, the original trials reported only the number of adverse events and did not provide information on event types, severity, causality, or methods of adverse event ascertainment. Consequently, the available evidence is insufficient to draw definitive conclusions regarding the safety of probiotic/synbiotic supplementation in patients with CHD, and its safety profile remains uncertain.

**Figure 6:**
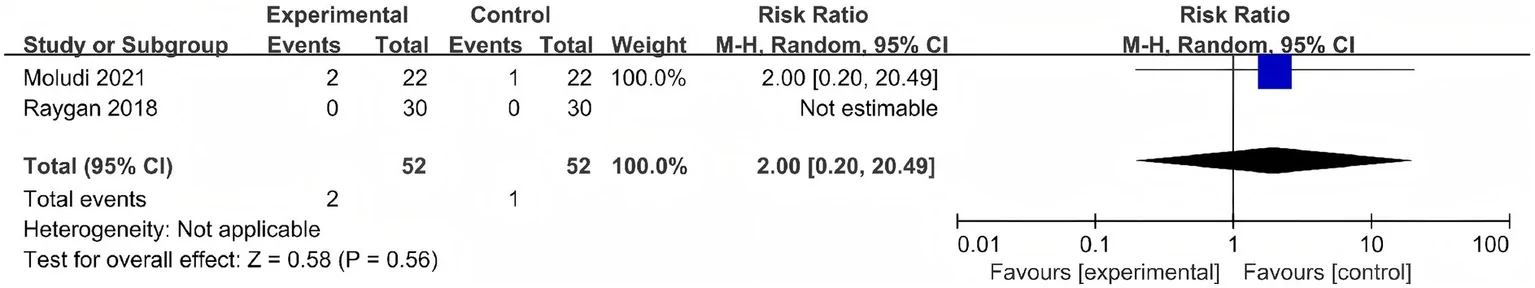
Forest plots of the meta-analysis on the adverse event rate.

### 3.5 Subgroup analysis

The initial meta-analysis showed substantial heterogeneity for LDL-C (*I*^2^ = 67%). Therefore, subgroup analyses were performed to investigate potential sources of clinical heterogeneity according to country, average age, intervention type, probiotic strain, probiotic dosage, and treatment duration, as summarized in Table 2. Given the small number of available studies, these subgroup analyses were considered exploratory and hypothesis-generating only.

**Table 2:** Subgroup analyses of LDL-C heterogeneity sources investigation.

Subject	Subgroup differences	Subgroup	Number of studies	*I*^2^/%	MD (95% CI)	*p*-value
Country	*p* = 0.0006, *I*^2^ = 91.5%	China	1	0	−18.95 (−22.75, −15.15)	<0.00001
		Iran	4	0	−4.21 (−11.73, 3.31)	0.27
Average age	*p* = 0.37, *I*^2^ = 0%	<60 years old	2	0	−7.44 (−26.17, 11.29)	0.44
		≥60 years old	3	82	−9.69 (−21.85, 2.47)	0.12
Intervention type	*p* = 0.01, *I*^2^ = 83.1%	Probiotic	3	66	−17.35 (−20.92, −13.78)	<0.00001
		Synbiotic	2	0	−3.24 (−14.02, 7.54)	0.56
Probiotic strain	*p* = 0.001, *I*^2^ = 90.5%	Single-strain	3	0	−18.50 (−22.22, −14.78)	<0.00001
		Multi-strain	2	0	−3.59 (−11.80, 4.63)	0.39
Probiotic dosage	*p* = 0.37, *I*^2^ = 0%	<2 × 10^9^ CFU/day	2	0	−7.44 (−26.17, 11.29)	0.44
		≥2 × 10^9^ CFU/day	3	82	−9.69 (−21.85, 2.47)	0.12
Treatment duration	*p* = 0.0006, *I*^2^ = 91.5%	≤12 weeks	4	0	−4.21 (−11.73, 3.31)	0.27
		24 weeks	1	0	−18.95 (−22.75, −15.15)	<0.00001

When stratified by country, probiotic/synbiotic supplementation was associated with a significant reduction in LDL-C levels among participants from China (MD − 18.95, 95% CI − 22.75 to −15.15, *p* < 0.00001, *I*^2^ = 0), whereas no significant effect was observed in participants from Iran (MD − 4.21, 95% CI − 11.73 to 3.31, *p* = 0.27, *I*^2^ = 0). The test for subgroup differences was statistically significant (*p* = 0.0006, *I*^2^ = 91.5%) (Supplementary Figure S2). However, the China subgroup comprised only one study, and therefore the within-subgroup heterogeneity statistic could not be meaningfully interpreted. The observed difference between subgroups should consequently be regarded as an exploratory finding rather than evidence that country explains the heterogeneity.

Similarly, significant subgroup differences were observed according to probiotic strain (*p* = 0.001, *I*^2^ = 90.5%). Probiotic/synbiotic supplementation was associated with a significant reduction in LDL-C levels in the single-strain subgroup (MD − 18.50 mg/dL, 95% CI − 22.22 to −14.78, *p* < 0.00001, *I*^2^ = 0), whereas no significant effect was found in the multi-strain subgroup (MD − 3.59 mg/dL, 95% CI − 11.80 to 4.63, *p* = 0.39, *I*^2^ = 0) (Supplementary Figure S3). These findings may indicate differences according to strain formulation, but given the limited number of studies within the subgroups, they should be interpreted cautiously and considered hypothesis-generating.

Significant subgroup differences were also identified according to treatment duration (*p* = 0.0006, *I*^2^ = 91.5%). Twenty-four weeks of probiotic/synbiotic supplementation was associated with a significant reduction in LDL-C levels (MD − 18.95 mg/dL, 95% CI − 22.75 to −15.15, *p* < 0.00001, *I*^2^ = 0), whereas no significant effect was observed for interventions lasting ≤12 weeks (MD − 4.21 mg/dL, 95% CI − 11.73 to 3.31, *p* = 0.27, *I*^2^ = 0) (Supplementary Figure S4). Because the 24-week subgroup comprised only one study, its within-subgroup *I*^2^ value was not considered informative. Thus, the observed difference according to treatment duration should be interpreted as an exploratory finding rather than evidence of a definite duration-dependent effect.

Notably, when stratified by intervention type, significant subgroup differences were also observed (*p* = 0.01, *I*^2^ = 83.1%). Probiotic supplementation was associated with a significant reduction in LDL-C levels (MD − 17.35 mg/dL, 95% CI − 20.92 to −13.78, *p* < 0.00001, *I*^2^ = 66%), whereas no significant association was observed for synbiotic supplementation (MD − 3.24 mg/dL, 95% CI − 14.02 to 7.54, *p* = 0.56, *I*^2^ = 0) (Supplementary Figure S5). However, because the synbiotic subgroup included only two studies, the absence of a statistically significant association should be interpreted cautiously and should not be considered evidence of a true difference in efficacy between probiotics and synbiotics.

No significant subgroup differences were identified according to average age or probiotic dosage. Overall, because only five studies contributed to the LDL-C analysis and several subgroup categories contained one or two studies, these analyses have limited statistical power and should be considered exploratory and hypothesis-generating rather than confirmatory. In particular, I^2^ estimates from subgroups containing only one study were not interpreted as evidence for the presence or absence of heterogeneity. The observed subgroup differences therefore require confirmation in future adequately powered RCTs.

### 3.6 Sensitivity analysis

To further investigate the source of heterogeneity and assess the robustness of the pooled estimates, leave-one-out sensitivity analyses were performed, as summarized in Table 3. For LDL-C, the leave-one-out analyses produced pooled effect estimates ranging from MD −12.28 to −4.21 mg/dL, with *p* values ranging from 0.01 to 0.09. The heterogeneity observed in the primary analysis was substantially reduced to I^2^ = 0% after excluding Sun et al. (2022), suggesting that this study may have contributed importantly to the observed heterogeneity. Notably, Sun et al. was the only study that included participants from China, whereas the other studies were conducted in Iran, suggesting that differences in study population or country may be a potential source of heterogeneity. However, this finding should be interpreted cautiously because only one study represented the Chinese population. In contrast, exclusion of Tajabadi-Ebrahimi et al. (2017) changed the statistical significance of the pooled effect, with LDL-C becoming significantly reduced (MD − 12.28 mg/dL, 95% CI − 21.91 to −2.66, *p* = 0.01). Thus, the LDL-C finding was considered non-robust and should be interpreted cautiously.

**Table 3:** Leave-one-out sensitivity analyses.

Outcome	Range of RR/MD	Range of *P*-value	Study influencing pooled estimate	*I*^2^/%	RR/MD (95% CI)	*P*-value	Robustness
LDL-C	−12.28 to −4.21	0.01–0.09	Tajabadi-Ebrahimi et al. (2017)	0	−12.28 (−21.91, −2.66)	0.01	Non-robust
VLDL-C	−2.40 to 0.60	0.48–0.86	None	–	–	–	Robust
HDL-C	2.67 to 4.16	0.0002–0.04	None	–	–	–	Robust
TC	−6.58 to −0.78	0.49–0.89	None	–	–	–	Robust
TG	−18.21 to −6.21	0.18–0.58	None	–	–	–	Robust
FBG	−20.94 to −18.13	0.006–0.03	None	–	–	–	Robust
Insulin	−4.30 to −1.60	0.07–0.38	None	–	–	–	Robust
HOMA-IR	−0.50 to −0.20	0.61–0.78	None	–	–	–	Robust
QUICKI	Maintain 0.00	Maintain 1.00	None	–	–	–	Robust
SBP	−4.85 to −3.44	0.13–0.25	None	–	–	–	Robust
DBP	−4.78 to −2.12	0.26–0.36	None	–	–	–	Robust
AER	Maintain 2.00	Maintain 0.56	None	–	–	–	Robust

RR, risk ratio; MD, mean difference; CI, confidence interval; LDL-C, low-density lipoprotein cholesterol; VLDL-C, very low-density lipoprotein cholesterol; HDL-C, high-density lipoprotein cholesterol; TC, total cholesterol; TG, triglycerides; FBG, fasting blood glucose; HOMA-IR, homeostasis model assessment of insulin resistance; QUICKI, quantitative insulin sensitivity check index; SBP, systolic blood pressure; DBP, diastolic blood pressure; AER, adverse event rate.

For the remaining outcomes, the leave-one-out analyses showed relatively stable pooled estimates. Specifically, VLDL-C (MD range −2.40 to 0.60 mg/dL, *p* = 0.48–0.86), HDL-C (MD range 2.67 to 4.16 mg/dL, *p* = 0.0002–0.04), TC (MD range −6.58 to −0.78 mg/dL, *p* = 0.49–0.89), TG (MD range −18.21 to −6.21 mg/dL, *p* = 0.18–0.58), FBG (MD range −20.94 to −18.13 mg/dL, *p* = 0.006–0.03), insulin (MD range −4.30 to −1.60 μIU/mL, *p* = 0.07–0.38), HOMA-IR (MD range −0.50 to −0.20, *p* = 0.61–0.78), SBP (MD range −4.85 to −3.44 mmHg, *p* = 0.13–0.25), and DBP (MD range −4.78 to −2.12 mmHg, *p* = 0.26–0.36). For QUICKI, the pooled estimate and *p* value remained unchanged (MD = 0.00, *p* = 1.00), as did those for AER (RR = 2.00, *p* = 0.56). Overall, the direction and statistical interpretation of these pooled estimates remained generally consistent after sequential exclusion of individual studies, supporting the robustness of these findings.

### 3.7 TSA

To avoid deriving the required information size (RIS) from the pooled effect estimates of the present meta-analysis, the anticipated intervention effects for HDL-C and FBG were determined independently from relevant previous literature. An anticipated MD of 1.28 mg/dL was used for HDL-C (Apisitwittaya et al., 2026) and 11.18 mg/dL for FBG (Naseri et al., 2022), while the variance was calculated from the corresponding trials included in the present meta-analysis. A random-effects model consistent with the primary meta-analysis was used. Because no statistical heterogeneity was observed for either HDL-C or FBG (*I*^2^ = 0%), the RIS was not further adjusted for diversity.

For HDL-C, the anticipated intervention effect was 1.28 mg/dL, and the variance was 52.17, yielding an RIS of 1,000 participants. The cumulative information did not reach the calculated RIS, as shown in Figure 7A. For FBG, the anticipated intervention effect was 11.18 mg/dL, and the variance was 120.98, yielding an RIS of 533 participants. The cumulative Z-curve did not cross the TSA monitoring boundary or reach the calculated RIS, as shown in Figure 7B.

**Figure 7:**
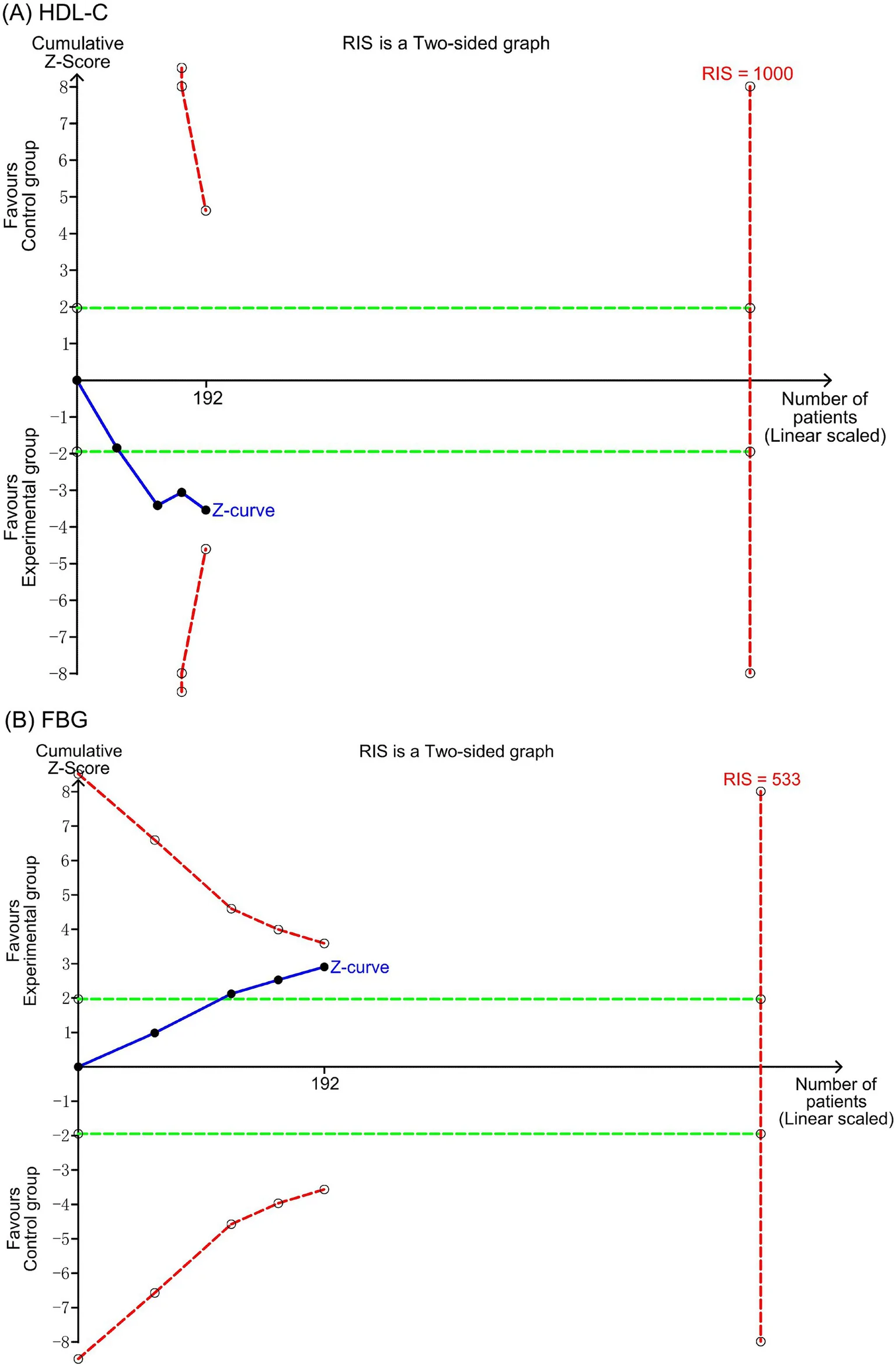
Trial sequential analyses of efficacy outcomes: **(A)** HDL-C; **(B)** FBG. HDL-C, high-density lipoprotein cholesterol; FBG, fasting blood glucose.

However, the TSA findings should be interpreted cautiously given the small number of available studies and potential clinical heterogeneity in geographic region, probiotic strains, and intervention duration. Further large-scale, multicenter RCTs are warranted to confirm these findings.

### 3.8 Publication bias

Publication bias was not assessed for any outcome because fewer than 10 studies were available per outcome. As a result, funnel plots and Egger’s tests were considered to have insufficient power and potentially unreliable, and were therefore not performed in accordance with the prespecified methodology.

### 3.9 Certainty of evidence

According to the GRADE framework, the certainty of evidence was rated as low for VLDL-C, HDL-C, TC, TG, FBG, insulin, HOMA-IR, QUICKI, SBP, and DBP, and very low for LDL-C and AER (Table 4). Overall, these ratings indicate substantial uncertainty regarding the magnitude and reliability of the observed effects. These findings underscore the need for larger, well-designed randomized controlled trials to strengthen the evidence base and clarify the effects of specific probiotic/synbiotic interventions on metabolic outcomes in patients with CHD.

**Table 4:** Certainty of evidence.

Outcome	Risk of bias	Inconsistency	Indirectness	Imprecision	Publication bias	RR/MD (95% CI)	Certainty of evidence
LDL-C	Serious^a^	Serious^b^	None	Very Serious^c^	Not assessed^f^	−9.50 (−19.17, 0.17)	Very Low
VLDL-C	Serious^a^	None	None	Serious^d^	Not assessed^f^	−0.93 (−5.70, 3.84)	Low
HDL-C	Serious^a^	None	None	Serious^d^	Not assessed^f^	3.70 (1.65, 5.74)	Low
TC	Serious^a^	None	None	Serious^d^	Not assessed^f^	−1.94 (−12.41, 8.52)	Low
TG	Serious^a^	None	None	Serious^d^	Not assessed^f^	−10.22 (−31.19, 10.75)	Low
FBG	Serious^a^	None	None	Serious^d^	Not assessed^f^	−19.25 (−32.28, −6.22)	Low
Insulin	Serious^a^	None	None	Serious^d^	Not assessed^f^	−2.60 (−5.43, 0.22)	Low
HOMA-IR	Serious^a^	None	None	Serious^d^	Not assessed^f^	−0.31 (−1.46, 0.84)	Low
QUICKI	Serious^a^	None	None	Serious^d^	Not assessed^f^	0.00 (−0.01, 0.01)	Low
SBP	Serious^a^	None	None	Serious^d^	Not assessed^f^	−4.25 (−9.63, 1.12)	Low
DBP	Serious^a^	None	None	Serious^d^	Not assessed^f^	−2.40 (−6.26, 1.47)	Low
AER	Serious^a^	None	None	Very Serious^e^	Not assessed^f^	2.00 (0.20, 20.49)	Very Low

^a^Downgraded one level for risk of bias because several studies had some concerns regarding the randomization process, missing outcome data, or selection of the reported result, and one study was judged to have a high overall risk of bias due to substantial missing outcome data. ^b^Downgraded one level for inconsistency because substantial between-study heterogeneity was observed (*I*^2^ = 67%). ^c^Downgraded two levels for very serious imprecision because the confidence interval crossed the null effect and encompassed substantially different plausible effects, ranging from potentially meaningful benefit to little or no benefit, as observed for LDL-C. ^d^Downgraded one level for imprecision because the available information was limited and/or the confidence interval indicated uncertainty in the estimated effect. ^e^Downgraded two levels for very serious imprecision because of the very small number of events and the extremely wide confidence interval, making the estimated effect highly uncertain, as observed for AER. ^f^Publication bias was not assessed because fewer than 10 studies were available for each outcome. RR, risk ratio; MD, mean difference; CI, confidence interval; LDL-C, low-density lipoprotein cholesterol; VLDL-C, very low-density lipoprotein cholesterol; HDL-C, high-density lipoprotein cholesterol; TC, total cholesterol; TG, triglycerides; FBG, fasting blood glucose; HOMA-IR, homeostasis model assessment of insulin resistance; QUICKI, quantitative insulin sensitivity check index; SBP, systolic blood pressure; DBP, diastolic blood pressure; AER, adverse event rate.

## 4 Discussion

### 4.1 Research significance and findings

CHD remains one of the leading causes of morbidity and mortality worldwide (GBD 2019 Diseases and Injuries Collaborators, 2020). Previous evidence has supported the potential of probiotic/synbiotic supplementation in regulating lipid metabolism, which lays a foundation for research in this field (Cho and Kim, 2015; Guo et al., 2011). With increasing insights into the “gut microbiota dysbiosis–glycolipid metabolism disorder–atherosclerosis” pathway (Tousoulis et al., 2022), probiotic/synbiotic supplementation has attracted considerable attention as a potential adjunctive therapy for CHD (Mirzahosseini et al., 2024). Our meta-analysis found that probiotic/synbiotic supplementation was associated with reductions in FBG and increases in HDL-C in patients with CHD. However, according to the GRADE assessment, the certainty of evidence ranged from low to very low, indicating that further well-designed studies are likely to substantially influence confidence in the estimated effects.

### 4.2 Efficacy and safety analyses

Abnormal lipid metabolism is one of the most critical risk factors and pathogenic mechanisms of CHD (Grundy et al., 2019). Our meta-analysis found that probiotic/synbiotic supplementation was associated with increases in HDL-C, with no significant effects on LDL-C, VLDL-C, TC, or TG. Furthermore, sensitivity analysis indicated that the HDL-C findings were generally stable, whereas TSA indicated that the cumulative evidence did not cross the monitoring boundary or reach the required information size. Moreover, GRADE rated the certainty of the evidence for HDL-C as low, reflecting limitations related to study quality, imprecision, and limited generalizability.

Notably, previous meta-analyses have reported inconsistent results regarding LDL-C. Lei et al. (2023) found that probiotic/synbiotic supplementation significantly reduced LDL-C (MD − 9.13 mg/dL, 95% CI − 13.17 to −5.09) and increased HDL-C (MD 1.94 mg/dL, 95% CI 0.32 to 3.57). Similarly, Dong et al. (2025) reported beneficial effects on LDL-C and HDL-C. One potential explanation for this discrepancy is the concurrent use of micronutrients, as both Lei et al. (2023) and Dong et al. (2025) included studies that combined probiotics/synbiotics with vitamin D or selenium, whereas our analysis focused exclusively on studies evaluating probiotic/synbiotic supplementation without concomitant micronutrient supplementation. Given the regulatory roles of vitamin D and selenium in lipid metabolism (Li et al., 2024; Zhang et al., 2023), these micronutrient co-interventions may have contributed to the lipid changes reported by Lei et al. (2023) and Dong et al. (2025), although this possibility cannot be confirmed from the available evidence. Additionally, the sensitivity analysis indicated that the pooled estimate for LDL-C was largely influenced by a single study, suggesting that the observed effect remains uncertain and should be interpreted cautiously until confirmed by additional high-quality RCTs. Therefore, current evidence is insufficient to conclude that probiotic/synbiotic supplementation reduces LDL-C levels in patients with CHD, and the positive findings reported by previous meta-analyses should be interpreted cautiously.

Although the meta-analysis suggested a potential improvement in HDL-C levels associated with probiotic/synbiotic supplementation, the cumulative evidence did not cross the TSA monitoring boundary or reach the required information size, and the certainty of evidence was rated as low. More importantly, an increase in HDL-C concentration does not necessarily translate into improved cardiovascular outcomes, and none of the included studies assessed hard clinical outcomes such as myocardial infarction, cardiovascular mortality, or major adverse cardiovascular events. Furthermore, given the heterogeneity in probiotic strains, combinations, doses, treatment durations, and intervention types across the included studies, these findings should not be generalized to all probiotics or synbiotics. Therefore, the potential benefit of probiotic/synbiotic supplementation on HDL-C remains uncertain, and the observed changes in HDL-C should not be interpreted as evidence of improved cardiovascular prognosis.

Disturbances in glucose metabolism are also key contributors to accelerated atherosclerosis and CHD progression (Poznyak et al., 2020). Our meta-analysis demonstrated that probiotic/synbiotic supplementation was associated with reductions in FBG levels but showed no significant association with insulin, HOMA-IR, or QUICKI. However, Lei et al. (2023) reported significant improvements in FBG (MD −13.60 mg/dL, 95% CI −23.57 to −3.62), insulin (MD − 3.39 μIU/mL, 95% CI −4.92 to −1.86), HOMA-IR (MD −0.98, 95% CI −1.63 to −0.31), and QUICKI (MD 0.02, 95% CI 0.01 to 0.03) with probiotic/synbiotic supplementation. This inconsistency may be attributable to differences in the included interventions and concomitant therapies, as Lei et al. (2023) included studies that combined probiotics with vitamin D or selenium. Vitamin D has been reported to improve glucose metabolism by preserving pancreatic β-cell function and alleviating oxidative stress, thereby mitigating insulin resistance (Szymczak-Pajor and Śliwińska, 2019; Argano et al., 2023). Similarly, selenium has been shown to protect β-cells from oxidative damage, leading to reductions in insulin and HOMA-IR levels (Ouyang et al., 2022). Thus, the reported benefits of probiotic/synbiotic supplementation on insulin, HOMA-IR, and QUICKI in Lei et al. (2023) may have been influenced by the concomitant use of vitamin D or selenium rather than probiotic/synbiotic supplementation alone, although this possibility cannot be confirmed from the available evidence.

Although the FBG finding in the present meta-analysis remained statistically significant and was generally stable in the sensitivity analysis, its reliability should be interpreted cautiously. TSA showed that the cumulative Z-curve did not cross the monitoring boundary or reach the required information size, indicating that the available cumulative evidence remains insufficient under the predefined TSA assumptions. Consistent with this finding, the certainty of evidence for FBG was rated as low according to GRADE. Therefore, the statistically significant reduction in FBG should be regarded as a potential metabolic effect rather than definitive evidence of a reliable treatment benefit. In addition, given the heterogeneity in probiotic strains, combinations, doses, and treatment durations across the included studies, these findings should not be generalized to all probiotics or synbiotics. Overall, probiotic/synbiotic supplementation may be associated with changes in FBG levels, but current evidence remains insufficient to support a clear benefit in glucose metabolism among patients with CHD.

Blood pressure is closely associated with CHD prognosis and mortality (van den Hoogen et al., 2000; Franklin et al., 2001). Our meta-analysis found that probiotic/synbiotic supplementation had no significant effects on SBP or DBP in patients with CHD. Sensitivity analysis confirmed the robustness of these findings. Similarly, the meta-analysis by Lei et al. (2023) reported consistent findings, showing no significant benefits of probiotic/synbiotic supplementation on SBP (MD −0.88 mmHg, 95% CI −3.72 to 1.96) or DBP (MD −0.21 mmHg, 95% CI −2.19 to 1.76), further supporting our results. Collectively, these findings indicate that probiotic/synbiotic supplementation was not associated with significant changes in blood pressure in the present analysis.

Regarding safety, although our meta-analysis found no statistically significant difference in AER between the probiotic/synbiotic supplementation group (2 cases, AER 3.8%) and the placebo group (1 case, AER 1.9%), this finding should not be interpreted as evidence of safety. Only two trials involving 104 participants reported adverse events, with very few events overall and a wide confidence interval for the pooled estimate, resulting in substantial uncertainty regarding the safety effect. Moreover, the original studies provided limited information on the types, severity, causality, and ascertainment of adverse events. Therefore, the available evidence is insufficient to adequately characterize the safety profile of probiotic/synbiotic supplementation in patients with CHD, and the safety of these interventions remains uncertain.

### 4.3 Discoveries and implications

This meta-analysis provides an updated assessment of the effects of probiotic/synbiotic supplementation on glycolipid metabolism in patients with CHD and provides additional evidence regarding the current uncertainties. Our findings indicate that probiotic/synbiotic supplementation was associated with increases in HDL-C and reductions in FBG, suggesting a potential role in modulating glycolipid metabolism among patients with CHD. However, unlike several recently published and earlier meta-analyses (Zhong et al., 2026; Yang et al., 2025; Lei et al., 2023; Dong et al., 2025), our results do not support a consistent benefit of probiotic/synbiotic supplementation on LDL-C, insulin levels, HOMA-IR, or QUICKI. These discrepancies may be attributable to several important methodological differences between the present study and previous reviews. First, Zhong et al. (2026) and Yang et al. (2025) included the trial by Moludi et al. (2019) involving patients with acute myocardial infarction. Because acute myocardial infarction is accompanied by profound inflammatory activation, metabolic disturbances, and intensive acute-phase medical management, inclusion of these patients may introduce additional clinical heterogeneity when evaluating glycolipid metabolic outcomes. In the present study, only patients with CHD who met our predefined eligibility criteria were included, thereby reducing potential clinical heterogeneity. Second, unlike several previous meta-analyses, including those by Lei et al. (2023) and Dong et al. (2025), our analysis excluded trials in which probiotic/synbiotic supplementation was administered together with micronutrients such as vitamin D or selenium. Since these micronutrients have independently been associated with improvements in lipid and glucose metabolism, their concomitant use may have contributed to larger estimated intervention effects reported in earlier reviews. By focusing on probiotic/synbiotic supplementation without additional micronutrient co-interventions, the present study provides an estimate that is less susceptible to this source of confounding. Finally, compared with previous meta-analyses, the present study places greater emphasis on the certainty and clinical interpretability of the evidence by integrating GRADE assessment, reassessment of cumulative evidence using TSA, evaluation of clinical relevance, and explicit consideration of important methodological limitations. These refinements provide a more cautious interpretation of the current evidence and may help reduce the risk of overestimating the therapeutic potential of specific probiotic/synbiotic interventions in CHD.

However, although statistically significant, the observed improvements in HDL-C and FBG should be interpreted cautiously with regard to their clinical relevance. Because no well-established CHD-specific MCIDs for HDL-C or FBG were identified, we used the MCID values reported in a previous systematic review and dose–response meta-analysis of cardiometabolic risk factors as reference thresholds. The pooled increase in HDL-C was 3.70 mg/dL, which was close to but slightly below the reported MCID of 3.86 mg/dL (Kiani et al., 2024). Similarly, the pooled reduction in FBG was 19.25 mg/dL, which did not reach the reported MCID of 28.82 mg/dL (Kiani et al., 2024). These comparisons suggest that the observed changes in HDL-C and FBG may be modest in relation to these reference thresholds. Importantly, these MCID values were derived from a population with cardiometabolic risk factors rather than specifically from patients with established CHD, and they were not developed in the context of probiotic/synbiotic supplementation. Therefore, they should not be regarded as validated CHD-specific MCIDs, but rather as reference values for contextualizing the magnitude of the observed metabolic changes. The applicability of these thresholds to patients with CHD receiving contemporary cardiovascular therapy remains uncertain. Moreover, none of the included studies reported hard cardiovascular outcomes such as myocardial infarction or cardiovascular mortality. Consequently, the current evidence does not support definitive conclusions regarding whether probiotic/synbiotic supplementation can translate these metabolic changes into meaningful improvements in long-term cardiovascular prognosis.

Notably, previous meta-analyses conducted in non-CHD metabolic populations, including individuals with prediabetes, type 2 diabetes, overweight, and obesity, have demonstrated that probiotic/synbiotic supplementation can significantly reduce various glycolipid metabolic parameters, such as HbA1c, FBG, fasting insulin, HOMA-IR, LDL-C, TC, TG, SBP, and DBP (Setayesh et al., 2025a; Setayesh et al., 2025b; Sefidgari-Abrasi et al., 2025). However, the observed effects were generally larger than those noted in CHD patients in our analysis. This discrepancy may result from differences in baseline metabolic status, concomitant medication use, or the severity of systemic inflammation and vascular dysfunction in CHD patients, which may influence the metabolic response to probiotic/synbiotic supplementation. These findings suggest that the metabolic response to specific probiotic/synbiotic interventions may vary depending on the underlying metabolic and cardiovascular characteristics of the population studied.

Taken together, the present findings suggest that specific probiotic/synbiotic interventions may be associated with changes in selected glycolipid parameters in patients with CHD, but the current evidence remains insufficient to support specific clinical recommendations. Further large-scale, multicenter RCTs evaluating both metabolic and cardiovascular outcomes are required to determine the true clinical significance of probiotic/synbiotic supplementation in CHD management.

### 4.4 Limitations and prospects

Several limitations of this meta-analysis should be acknowledged. First, only six RCTs involving a total of 296 participants were included, and several outcomes were based on only two studies. Such limited evidence reduces the precision and robustness of the pooled estimates, and the findings should therefore be interpreted cautiously. In particular, outcomes based on only two studies, including VLDL-C, insulin, HOMA-IR, QUICKI, and AER, remain highly uncertain and require further validation in additional well-designed trials.

Second, the available evidence lacked geographical diversity, with five of the six included studies conducted in Iran and only one conducted in China. This geographic concentration substantially limits the external validity and generalizability of the findings. Differences in genetic background, dietary habits, gut microbiota composition, healthcare systems, and standard CHD management across regions may influence the response to probiotic/synbiotic supplementation and may also contribute to differences in metabolic outcomes. Consequently, the current evidence primarily reflects populations from the Middle East and East Asia and cannot be readily extrapolated to populations with different ethnic backgrounds, dietary patterns, microbiome profiles, or healthcare settings without further validation.

Third, the included studies evaluated heterogeneous interventions, including different probiotic strains, strain combinations, formulations (probiotics and synbiotics), dosages, and treatment durations. Because the effects of probiotics may be strain- and formulation-specific, pooling these interventions may have introduced biological heterogeneity. Therefore, the observed metabolic changes should not be interpreted as a general effect of all probiotics or synbiotics and may instead reflect the effects of particular strains or formulations evaluated in the included trials. Owing to the limited number of available studies, strain-specific or formulation-specific analyses were not feasible in the present meta-analysis. Future studies should directly compare different strains and formulations and provide sufficient information to facilitate more informative strain-specific meta-analyses.

Fourth, the intervention durations in most included studies ranged from 8 to 12 weeks, which may be insufficient to determine whether the observed metabolic changes can be sustained over time or whether they translate into meaningful long-term cardiovascular benefits. Longer intervention and follow-up periods are therefore needed to evaluate the durability of metabolic effects and their potential relationship with cardiovascular outcomes.

Fifth, although HDL-C was significantly increased in the present meta-analysis, an increase in HDL-C concentration does not necessarily translate into improved cardiovascular outcomes. None of the included studies assessed hard clinical outcomes such as myocardial infarction, cardiovascular mortality, or major adverse cardiovascular events. Thus, whether the observed metabolic changes translate into clinically meaningful improvements in cardiovascular prognosis remains uncertain.

Given these limitations, future studies should conduct large-scale, multicenter RCTs involving more diverse ethnic and geographic populations and longer intervention and follow-up periods to improve the generalizability and reliability of the evidence. Future trials should also standardize probiotic/synbiotic intervention protocols and comprehensively report strain characteristics, formulations, dosages, concomitant therapies, and safety outcomes to facilitate more informative strain- and formulation-specific analyses. Importantly, trials should incorporate clinically relevant cardiovascular outcomes, in addition to metabolic biomarkers, to determine whether changes in HDL-C, FBG, or other metabolic parameters translate into meaningful cardiovascular benefits.

## 5 Conclusion

Probiotic/synbiotic supplementation may be associated with an increase in HDL-C and a reduction in FBG levels in patients with CHD. However, the cumulative evidence for both outcomes did not cross the TSA monitoring boundaries or reach the required information sizes, and the certainty of evidence was rated as low. Moreover, changes in HDL-C or FBG should not be interpreted as evidence of clinically meaningful cardiovascular benefit, as none of the included studies evaluated hard cardiovascular outcomes. It is important to note that generalizability is limited, as five of the six studies were conducted in Iran and only one in China. This geographical concentration raises concerns about the applicability of the findings to populations with different dietary patterns, microbiome profiles, ethnic backgrounds, or healthcare systems. Given the substantial heterogeneity in probiotic strains, combinations, formulations, dosages, and treatment durations, these findings should not be generalized to all probiotics or synbiotics. Therefore, the current evidence is insufficient to support a definitive metabolic benefit of probiotic/synbiotic supplementation in patients with CHD. Further large-scale, multicenter RCTs involving diverse populations, standardized intervention protocols, longer follow-up, and clinically relevant cardiovascular outcomes are warranted.

## Data Availability

The original contributions presented in the study are included in the article/Supplementary material, further inquiries can be directed to the corresponding authors.
