# Supplementary material for: Effects of probiotic or synbiotic supplementation on glycolipid metabolism in patients with coronary heart disease: a systematic review and meta-analysis
Source: Front Microbiol. 2026 Oct 23;17:1877366. doi: 10.3389/fmicb.2026.1877366 (PMC13741933; doi:10.3389/fmicb.2026.1877366)
Supplement: Supplementary file 1 [file Table_1.DOCX]

**Supplementary Table S1** Search strategies and results for all databases.

| Database | Search strategy | Dates | Filters | Results |
| --- | --- | --- | --- | --- |
| PubMed | (Coronary Disease[Title/Abstract] OR Coronary Diseases[Title/Abstract] OR Coronary Heart Disease[Title/Abstract] OR Coronary Heart Diseases[Title/Abstract] OR Coronary Artery Disease[Title/Abstract] OR Coronary Artery Diseases[Title/Abstract] OR Myocardial Infarction[Title/Abstract] OR Heart Attack[Title/Abstract] OR Heart Attacks[Title/Abstract] OR Myocardial Infarct[Title/Abstract] OR Myocardial Infarcts[Title/Abstract]) AND (Probiotic[Title/Abstract] OR Probiotics[Title/Abstract] OR Synbiotic[Title/Abstract] OR Synbiotics[Title/Abstract] OR Bifidobacterium[Title/Abstract] OR Bifidobacteria[Title/Abstract] OR Bacillus bifida[Title/Abstract] OR Yeast[Title/Abstract] OR Saccharomyces cerevisiae[Title/Abstract] OR Saccharomyces italicus[Title/Abstract] OR Saccharomyces oviformis[Title/Abstract] OR S cerevisiae[Title/Abstract] OR S. cerevisiae[Title/Abstract] OR Saccharomyces uvarum var melibiosus[Title/Abstract] OR Candida robusta[Title/Abstract] OR Saccharomyces capensis[Title/Abstract] OR Lactobacillus acidophilus[Title/Abstract] OR Lactobacillus amylovorus[Title/Abstract] OR Lactobacill[Title/Abstract] OR lactic acid bacteria[Title/Abstract] OR Clostridium butyricum[Title/Abstract] OR Bacillus[Title/Abstract] OR Natto Bacteria[Title/Abstract] OR Streptococcus thermophiles[Title/Abstract] OR Enterococcus[Title/Abstract]) | May 1, 2026 | - | 420 |
| Embase | (Coronary Disease:ab,ti OR Coronary Diseases:ab,ti OR Coronary Heart Disease:ab,ti OR Coronary Heart Diseases:ab,ti OR Coronary Artery Disease:ab,ti OR Coronary Artery Diseases:ab,ti OR Myocardial Infarction:ab,ti OR Heart Attack:ab,ti OR Heart Attacks:ab,ti OR Myocardial Infarct:ab,ti OR Myocardial Infarcts:ab,ti) AND (Probiotic:ab,ti OR Probiotics:ab,ti OR Synbiotic:ab,ti OR Synbiotics:ab,ti OR Bifidobacterium:ab,ti OR Bifidobacteria:ab,ti OR Bacillus bifida:ab,ti OR Yeast:ab,ti OR Saccharomyces cerevisiae:ab,ti OR Saccharomyces italicus:ab,ti OR Saccharomyces oviformis:ab,ti OR S cerevisiae:ab,ti OR S. cerevisiae:ab,ti OR Saccharomyces uvarum var melibiosus:ab,ti OR Candida robusta:ab,ti OR Saccharomyces capensis:ab,ti OR Lactobacillus acidophilus:ab,ti OR Lactobacillus amylovorus:ab,ti OR Lactobacill:ab,ti OR lactic acid bacteria:ab,ti OR Clostridium butyricum:ab,ti OR Bacillus:ab,ti OR Natto Bacteria:ab,ti OR Streptococcus thermophiles:ab,ti OR Enterococcus:ab,ti) | May 1, 2026 | - | 647 |
| Cochrane Library | Coronary Disease OR Coronary Diseases OR Coronary Heart Disease OR Coronary Heart Diseases OR Coronary Artery Disease OR Coronary Artery Diseases OR Myocardial Infarction OR Heart Attack OR Heart Attacks OR Myocardial Infarct OR Myocardial Infarcts in Title Abstract Keyword AND Probiotic OR Probiotics OR Synbiotic OR Synbiotics OR Bifidobacterium OR Bifidobacteria OR Bacillus bifida OR Yeast OR Saccharomyces cerevisiae OR Saccharomyces italicus OR Saccharomyces oviformis OR S cerevisiae OR S. cerevisiae OR Saccharomyces uvarum var melibiosus OR Candida robusta OR Saccharomyces capensis OR Lactobacillus acidophilus OR Lactobacillus amylovorus OR Lactobacill OR lactic acid bacteria OR Clostridium butyricum OR Bacillus OR Natto Bacteria OR Streptococcus thermophiles OR Enterococcus in Title Abstract Keyword | May 1, 2026 | - | 144 |
| Web of Science | Coronary Disease OR Coronary Diseases OR Coronary Heart Disease OR Coronary Heart Diseases OR Coronary Artery Disease OR Coronary Artery Diseases OR Myocardial Infarction OR Heart Attack OR Heart Attacks OR Myocardial Infarct OR Myocardial Infarcts (Topic) and Probiotic OR Probiotics OR Synbiotic OR Synbiotics OR Bifidobacterium OR Bifidobacteria OR Bacillus bifida OR Yeast OR Saccharomyces cerevisiae OR Saccharomyces italicus OR Saccharomyces oviformis OR S cerevisiae OR S. cerevisiae OR Saccharomyces uvarum var melibiosus OR Candida robusta OR Saccharomyces capensis OR Lactobacillus acidophilus OR Lactobacillus amylovorus OR Lactobacill OR lactic acid bacteria OR Clostridium butyricum OR Bacillus OR Natto Bacteria OR Streptococcus thermophiles OR Enterococcus (Topic) | May 1, 2026 | - | 763 |
| MEDLINE | Coronary Disease OR Coronary Diseases OR Coronary Heart Disease OR Coronary Heart Diseases OR Coronary Artery Disease OR Coronary Artery Diseases OR Myocardial Infarction OR Heart Attack OR Heart Attacks OR Myocardial Infarct OR Myocardial Infarcts (Topic) and Probiotic OR Probiotics OR Synbiotic OR Synbiotics OR Bifidobacterium OR Bifidobacteria OR Bacillus bifida OR Yeast OR Saccharomyces cerevisiae OR Saccharomyces italicus OR Saccharomyces oviformis OR S cerevisiae OR S. cerevisiae OR Saccharomyces uvarum var melibiosus OR Candida robusta OR Saccharomyces capensis OR Lactobacillus acidophilus OR Lactobacillus amylovorus OR Lactobacill OR lactic acid bacteria OR Clostridium butyricum OR Bacillus OR Natto Bacteria OR Streptococcus thermophiles OR Enterococcus (Topic) | May 1, 2026 | - | 620 |
| ClinicalTrials.gov | (Coronary Disease OR Coronary Diseases OR Coronary Heart Disease OR Coronary Heart Diseases OR Coronary Artery Disease OR Coronary Artery Diseases OR Myocardial Infarction OR Heart Attack OR Heart Attacks OR Myocardial Infarct OR Myocardial Infarcts) AND (Probiotic OR Probiotics OR Synbiotic OR Synbiotics OR Bifidobacterium OR Bifidobacteria OR Bacillus bifida OR Yeast OR Saccharomyces cerevisiae OR Saccharomyces italicus OR Saccharomyces oviformis OR S cerevisiae OR S. cerevisiae OR Saccharomyces uvarum var melibiosus OR Candida robusta OR Saccharomyces capensis OR Lactobacillus acidophilus OR Lactobacillus amylovorus OR Lactobacill OR lactic acid bacteria OR Clostridium butyricum OR Bacillus OR Natto Bacteria OR Streptococcus thermophiles OR Enterococcus) | May 1, 2026 | - | 41 |

MEDLINE, Medical Literature Analysis and Retrieval System Online.
